# Investigation of electronic properties of graphene/Si field-effect transistor

**DOI:** 10.1186/1556-276X-7-677

**Published:** 2012-12-17

**Authors:** Xiying Ma, Weixia Gu, Jiaoyan Shen, Yunhai Tang

**Affiliations:** 1 School of Mathematics and Physics, Suzhou University of Science and Technology, 1# Kerui Road of Gaoxin Section, Suzhou, Jiangsu 215011, China

**Keywords:** Graphene/Si field-effect transistor, CVD, Current saturation, Local graphene gate

## Abstract

We report a high-performance graphene/Si field-effect transistor fabricated via rapid chemical vapor deposition. Oligolayered graphene with a large uniform surface acts as the local gate of the graphene transistors. The scaled transconductance, *g*_m_, of the graphene transistors exceeds 3 mS/μm, and the ratio of the current switch, *I*_on_/*I*_off_, is up to 100. Moreover, the output properties of the graphene transistor show significant current saturation, and the graphene transistor can be modulated using the local graphene gate. These results clearly show that the device is well suited for analog applications.

## Background

Graphene is a single-atom-thick carbon film [1,2] that has a very high carrier mobility (2 × 10^5^ cm^2^ V^−1^ s^−1^), a high saturation velocity, large current density, and thermal conductivity; as a result, it has attracted significant attention for use in high-speed applications and flexible electronics and as a candidate for next-generation technologies that enhance transistor performance beyond dimensional scaling [3,4]. To date, graphene-based electronics, including graphene field-effect transistors (GFETs) [5,6], nanoelectromechanical systems [7], molecular sensors [8], graphene-based luminescent diodes [9], and solar cells [10,11], have been reported. The simplest and most common approach for the fabrication of GFETs is to borrow mature microelectronic technology. This technology requires the deposition of large and uniform graphene thin films on a Si substrate in order to form a back gate. However, it is a challenge to synthesize low-defect and structurally continuous graphene mono- or oligolayers; this represents a major limitation for the rapid adoption of high-quality GFET applications. Graphene can be deposited on Si using the widely studied stripped method [12], cut-and-choose transfer printing [13], the epitaxial method [6,14], chemical vapor deposition (CVD) [15], and other methods [16,17]. The former two methods are very complex and increase the risk of impurities, which negatively affect transistor performance. Herein, for the rapid preparation of high-quality graphene films and GFETs, we adopt a low-pressure, rapid CVD technology. The surface morphology, structure, carrier concentration, and carrier mobility of the resultant graphene films are systemically studied. In addition, the transport properties of the GFET, such as transconductance, *g*_*m*_, ratio of current switch, *I*_on_/*I*_off_, and current saturation characteristics, are analyzed. Finally, the carrier transport mechanism in the GFET is discussed.

## Methods

**Figure 1:**
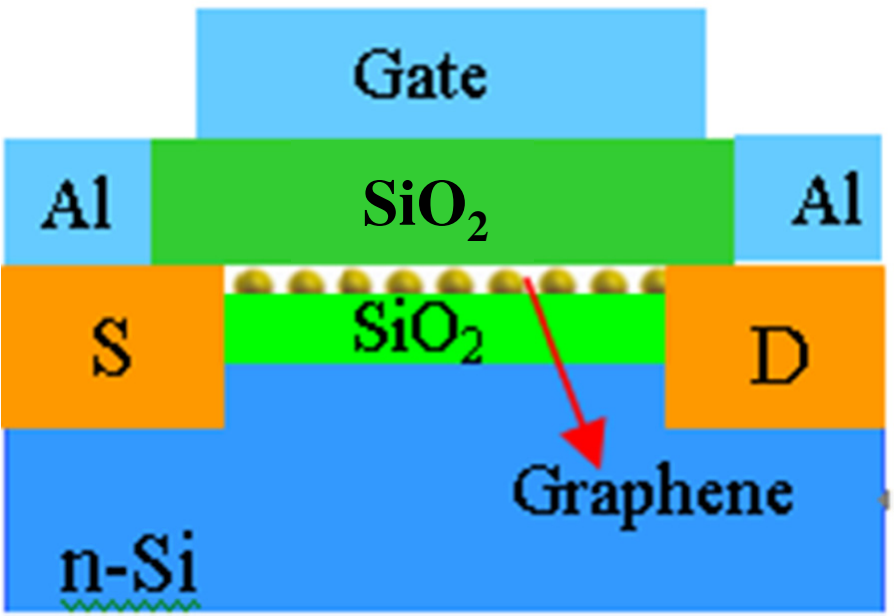
Schematic illustration of the graphene/Si field-effect transistor.

Figure 1 illustrates the structure of the graphene/Si field-effect transistors. Graphene acts as a local gate positioned on a highly doped n-type Si substrate separated by a 300-nm-thick thermal layer of silicon oxide. The oligolayered graphene film was fabricated using a rapid chemical vapor deposition process. The growth system comprises a large horizontal quartz tube furnace, a vacuum system, a gas meter, and a temperature controller. The n-type Si(100) substrates were ultrasonically cleaned with a sequence of acetone, ethanol, and deionized water, then dried with flowing N_2_, and placed at the center of the furnace. Prior to deposition, the furnace was evacuated to 10^−2^ Pa, heated to 300°C, and maintained at this temperature for 30 min to remove any moisture from the mixture. The furnace was then heated to 700°C for the deposition. A 300-μm SiO_2_ layer was thermally oxidized for 30 min using high-purity oxygen gas followed by annealing at 900°C for 40 min. The source and drain sections were formed by doping the Si with B atoms via exposure to an Ar gas flow carrying an analytically pure C_3_H_9_BO_3_ doping agent into the reactive chamber at 900°C for 20 min, followed by annealing at 950°C for 30 min. After doping, a mixture of CH_4_ gas (99.999%) and Ar gas at a volume ratio of 1:10 was introduced into the reaction chamber at the same temperature (950°C). CH_4_ decomposed to give a mixture of C, H^+^, and H_2_ when the vapor-phase CH_4_ and Ar gases flowed through the furnace; the C atoms condensed on the Si substrates to form the graphene film under a working pressure of 50 Pa. The growth process was carried out for 2 min, and the samples were then annealed at 1,000°C for 30 min. To observe the surface morphology and structure of the graphene film, a few graphene reference samples were concurrently deposited. Subsequently, the graphene film was capped with a 300-μm SiO_2_ layer, and the graphene and SiO_2_ layers above the source and drain sections were removed. Finally, the GFET was completed by depositing Al electrodes on the gate, source, and drain sections via evaporation. The morphologies and structures of the samples were characterized using optical microscopy, atomic force microscopy (AFM), and Raman spectroscopy. The electronic properties were assessed via Hall effect measurement (HMS-3000) and micro-current (4200, Keithley, Cleveland, OH, USA) measurements.

## Results and discussion

**Figure 2:**
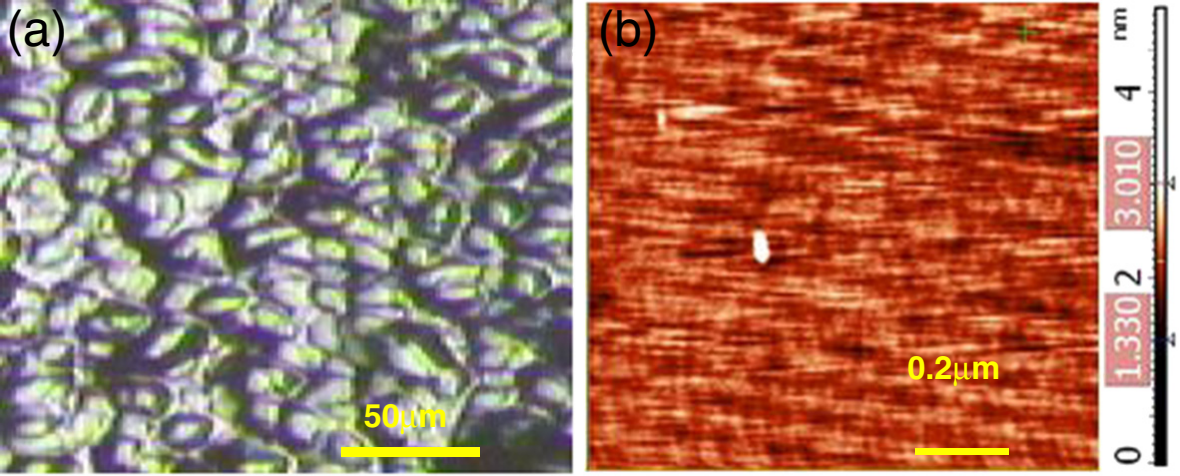
**Graphene deposited on the Si substrate.** (**a**) Optical microscopy image of the graphene deposited on the Si substrate, and (**b**) the enlarged AFM picture.

Figure 2a shows an optical microscopy image of the graphene deposited on the Si substrate. It is evident that many graphene slices or islands of about 10 μm in diameter are uniformly scattered on the substrate. Figure 2b is an enlarged picture of the graphene film obtained via AFM. The large-area graphene film has a smooth and uniform surface free of carbon clusters. The film is about 2-nm thick, which is equal to a few layers of graphene. From Figure 2, we can confirm that the growth of the graphene film is characteristic of the layer-island mode. C atoms from the decomposition of CH_4_ at 950°C (the decomposition temperature of CH_4_ is about 900°C) absorbed, condensed, and formed islands on the Si substrate. The graphene film was formed by the combination of many islands over time.

**Figure 3:**
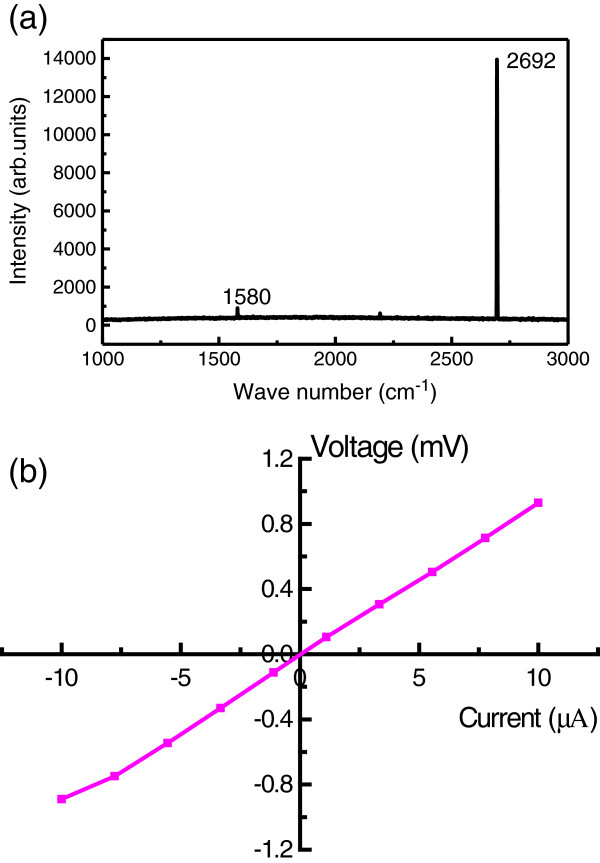
**Properties of the graphene film.** (**a**) Raman spectrum of the graphene film and (**b**) the surface voltage–current properties of the graphene film.

Figure 3a shows the Raman spectrum of the graphene film, which contains two major scattering peaks: a 2D-band peak at 2,690 cm^−1^ and a G-band peak at 1,590 cm^−1^. The intensity ratio of the peaks (*I*_2D_/*I*_G_) is 14, which confirms that the graphene film comprises a few layers and is of high quality. The surface carrier concentration, carrier mobility, and current–voltage (*I-V*) properties of the graphene film were determined via Hall effect measurements. The carrier concentration of the samples is about 10^10^ cm^−2^, while the electron mobility is 5.1 × 10^4^ cm^2^ V^−1^ s^−1^, which is very close to the known ideal value of 2 × 10^5^ cm^2^ V^−1^ s^−1^[3,4]. The surface *I-V* behavior of the graphene film is shown in Figure 3b. Clearly, the graphene surface shows a linear current–voltage relationship, which suggests good transport of electrons on the graphene film. For the Hall effect measurements, four measuring pole points are arranged in a square on the graphene surface. The *I-V* behaviors presented in Figure 3b indicate the relationships between the measured points. Since graphene film is a highly conductive material [18], electrons in graphene have high mobility. The voltage between two points changes in a linear relationship with the applied current, which closely obeys Ohm’s law.

**Figure 4:**
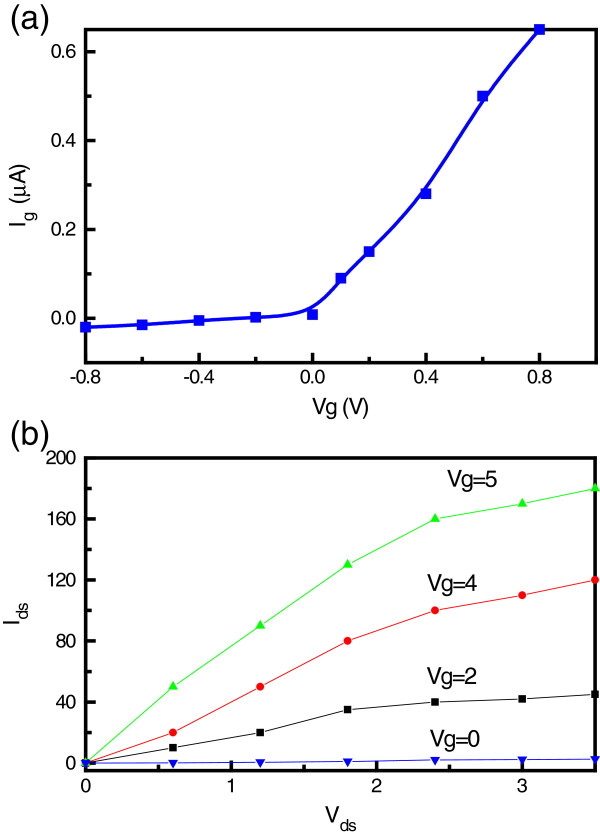
**The current–voltage behavior of the graphene/Si transistor.** (**a**) Gate current, *I*_g_, versus gate voltage, *V*_g_, behavior of the graphene/Si transistor and (**b**) the source-drain current, *I*_ds_, versus the source-drain voltage, *V*_ds_, with different gate voltages.

The gate current, *I*_g_, versus gate voltage, *V*_g_, behavior of the graphene/Si transistor is shown in Figure 4a. Remarkably, the device shows good rectification properties. The current increases exponentially with the applied positive voltage but is almost zero under the revised voltage. This shows that the GFET device does not have any significant gate leakage. Figure 4b shows the drain current, *I*_ds_, versus the source-drain voltage, *V*_ds_, under different gate voltages; the GFET exhibits a nearly linear plot when *V*_ds_ is less than 2.5 V for different gate voltages. As *V*_ds_ increases above 2.5 V, *I*_ds_ tends towards saturation. The results indicate that the *IV* plot for a *V*_g_ of 4 V is optimal as a ratio of current switch, *I*_on_/*I*_off_, of 100 is obtained. Moreover, the GFET has a smaller threshold voltage, and the *I*_ds_*-V*_ds_ plots of the graphene transistor show significant current saturation characteristics, which is typical of Si FETs but rare for GFETs. It has been suggested that the velocity saturation of the carrier at higher biases may lead to the current saturation phenomenon in graphene transistors [19]. The saturation velocity depends on the charge carrier concentration and scattering by interfacial phonons in the SiO_2_ layer that supports the graphene channels [20,21]. The achieved current saturation of the GFET makes this device well suited for analog applications. These results demonstrate the feasibility of two-dimensional graphene devices for analog and radio-frequency circuit applications without the need for bandgap engineering.

**Figure 5:**
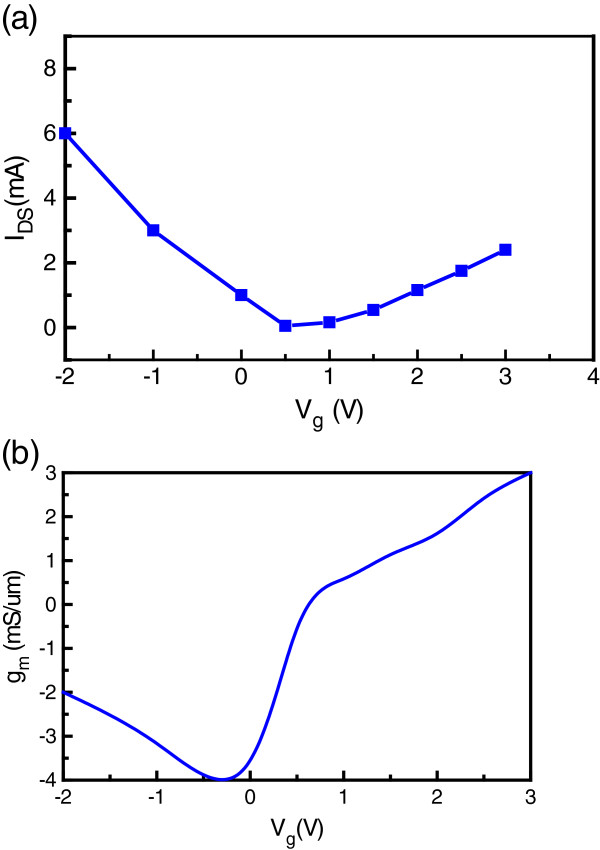
**The drain-source current, *I ***_**ds**_**, versus top-gate voltage (a), *V ***_**g**_**, and the electron transconductance, *g ***_**m**_**(b).**

Moreover, we determined the transfer characteristics of *I*_ds_ versus *V*_g_ at a *V*_ds_ value of 1 V (Figure 5a). The plot clearly shows that the graphene transistor can be modulated via the local graphene gate within the range of −2 to 3.5 V, which demonstrates that graphene can act as an effective gate for graphene transistors. Figure 5b shows the measured electron transconductance, *g*_m_ (where *g*_m_ = *∂I*_ds_/*∂V*_ds_), of the GFET as a function of the gate voltage. A peak transconductance of 3 mS/μm was obtained at a *V*_ds_ value of 1 V in this device. Significantly, this scaled transconductance is nearly one order of magnitude better than that of the reported graphene transistors [22]. The sign and magnitude of *g*_m_ is strongly dependent on the gate voltage. The branch where *g*_m_ is less than zero represents p-type transport that is dominated by holes; when *g*_m_ becomes positive with increasing gate voltage, the channel becomes n-type.

## Conclusions

High-performance graphene/Si transistors were fabricated by rapid chemical vapor deposition. The electron mobility of the graphene film is 5.1 × 10^4^ cm^2^ V^−1^ s^−1^, the scaled transconductance of the graphene transistors exceeds 3 mS/μm, and the ratio of the current switch, *I*_on_/*I*_off_, is as high as 100. The fabricated GFET shows current saturation characteristics which demonstrate that two-dimensional graphene devices can be used for analog and radio-frequency circuit applications without the need for bandgap engineering.

## Competing interests

The authors declare that they have no competing interests.

## Authors’ contributions

XM designed the structure of the graphene transistor, analyzed the results, and wrote the manuscript. WG participated in the fabrication of the graphene film on the substrate. JS fabricated the drain, source, and gate of the transistor and participated in the analysis the results of the transistor. YT measured the electrical properties of the transistor. All authors read and approved the final manuscript.

## Authors’ information

XM is a professor and PhD degree holder specializing in semiconductor materials and devices, specially expert in nanoscaled optical-electronic materials and optoelectronic devices. WG is a graduate student major in fabrication of new semiconductor nanometer materials. JS is a lecturer and PhD degree holder specializing in semiconductor devices. YT is an engineer specializing in optoelectronic measurements.
